# Minimally Invasive Colorectal Surgery Techniques

**DOI:** 10.7759/cureus.47203

**Published:** 2023-10-17

**Authors:** Akash Pathak, Mayur Wanjari

**Affiliations:** 1 Community Medicine, Jawaharlal Nehru Medical College, Datta Meghe Institute of Higher Education and Research, Wardha, IND; 2 Research and Development, Jawaharlal Nehru Medical College, Datta Meghe Institute of Higher Education and Research, Wardha, IND

**Keywords:** laparoscopy, robotic, non invasive, colorectal, surgery

## Abstract

Colorectal surgery has witnessed a transformative shift with the advent of minimally invasive techniques, offering patients reduced postoperative discomfort, shorter hospital stays, and accelerated recovery compared to conventional open surgery. This comprehensive review aims to assess the current state of minimally invasive approaches in colorectal surgery, encompassing various techniques such as single-incision laparoscopic surgery, robot-assisted surgery, and conventional laparoscopic surgery. The article meticulously explores the benefits and drawbacks of each technique, delves into the established criteria for their application, delineates cautious circumstances, and analyzes the outcomes of minimally invasive colorectal surgery. Additionally, the integration of virtual reality and augmented reality for surgical planning and training is discussed, shedding light on the future trajectory of this field. Surgeons and researchers striving to enhance patient care and surgical outcomes in colorectal surgery will find this review article an invaluable resource, presenting crucial components of minimally invasive colorectal surgery and paving the way for continued advancements in the field.

## Introduction and background

Over the past three decades, surgical innovation has been focused on the desire to lessen surgical stress and improve endoscopic access. Since Semm performed the first laparoscopic appendectomy in 1981, minimally invasive colon surgery has seen remarkable progress. Laparoscopic and minimally invasive surgery have been greatly advanced by recent developments in video and robotic technology [1]. The robotic technique is extremely helpful for medium and inferior rectal tumors, especially in males with a small pelvis and those with a high body mass index. The laparoscopic approach is highly effective for colon and superior rectal malignancies. Robotic methods help make the minimally invasive procedure easier in these situations, frequently protecting the pelvic autonomic nerves and improving quality of life. The surgical treatment for rectal cancer is a fascinating topic, especially in light of the different endpoints that are used to evaluate results and quality [2]. Quality of life and overall survival are important objectives for the treatment of rectal cancer. Due to its softer intra-abdominal tissue manipulation, the robotic method among minimally invasive techniques looks to be less invasive than traditional laparoscopic surgery (LS) and hand-assisted techniques. Reduced surgical stress response, which eventually results in decreased morbidity, might be the cause of the possible therapeutic advantages of minimally invasive rectal cancer therapies. An overview of the present status of minimally invasive surgery and the various developments over the previous 20 years will be given in this article. We must make sure that the quality of surgical results remains a top priority even when new surgical instruments and procedures are developed.

A lengthy process spanning three decades has led to the examination and implementation of minimally invasive surgery for colorectal cancer, particularly for rectal cancer. Due to its intricacy involving many quadrants and early worries about a greater frequency of port site recurrences, colon cancer surgery offered hurdles in contrast to procedures like gallbladder and appendectomy. Prior to the publication of adequately planned trials in the mid-2000s, laparoscopic procedures were thoroughly assessed for colon cancer through a number of non-randomized observational studies. Notably, individuals with rectal cancer were frequently omitted from these studies. Randomized controlled trials (RCTs) contrasting laparoscopic and open surgery for colon cancer, including the Barcelona, Clinical Outcomes of Surgical Therapy (COST), Colon Cancer Laparoscopic or Open Resection (COLOR), and Conventional vs. Laparoscopic-Assisted Surgery in Patients With Colorectal Cancer (CLASICC) trials, consistently showed at least equivalent oncologic outcomes, comparable complications, a modestly shorter hospital stay, and the restoration of bowel function following laparoscopic surgery [3]. But despite the early resistance, laparoscopic surgery grew common and had quantifiable advantages.

The Medical Research Council's (MRC) CLASICC experiment, which included patients with both colon and rectal cancer, featured the first randomized controlled trial contrasting laparoscopic versus open surgery for rectal cancer [4]. There were concerns regarding laparoscopic surgery for rectal cancer at the time due to a non-significant rise in positive circumferential resection margins (CRM) in the rectal arm. A big randomized experiment wasn't finished for another five years [5]. Similar short-term results were found for the two modalities in the South Korean Comparison of Open versus Laparoscopic Surgery for Mid- or Low-REctal Cancer After Neoadjuvant Chemoradiotherapy (COREAN) experiment. In patients undergoing laparoscopic surgery, the COLOR II study in Europe showed a speedier restoration of bowel function and much shorter hospital stays, with the same primary outcome of locoregional recurrence (5% in each group) [6]. The development of robotic rectal cancer surgery as a third technical modality during the early 2000s coincided with advancements in the validation of laparoscopy and the execution of the aforementioned trials [7]. Although initial reservations about laparoscopy-like issues such as longer operating times, probable oncological inferiority, and greater costs were voiced, robotic surgery quickly gained acceptance. Prior to the 2017 release of the first prospective randomized multicenter study, the majority of the data were observational [8]. Conversion rates were the main outcome of the Robotic-Assisted vs Conventional Laparoscopic Surgery on Risk of Conversion to Open Laparotomy Among Patients Undergoing Resection for Rectal Cancer (ROLARR) experiment, a worldwide undertaking. Except in subgroup analysis, there were sadly no significant differences between the two modes. A later, smaller RCT from Korea that used the total mesorectal excision (TME) specimen's quality as its main outcome also revealed comparable outcomes across the two groups [9].

## Review

Methodology

**Figure 1:**
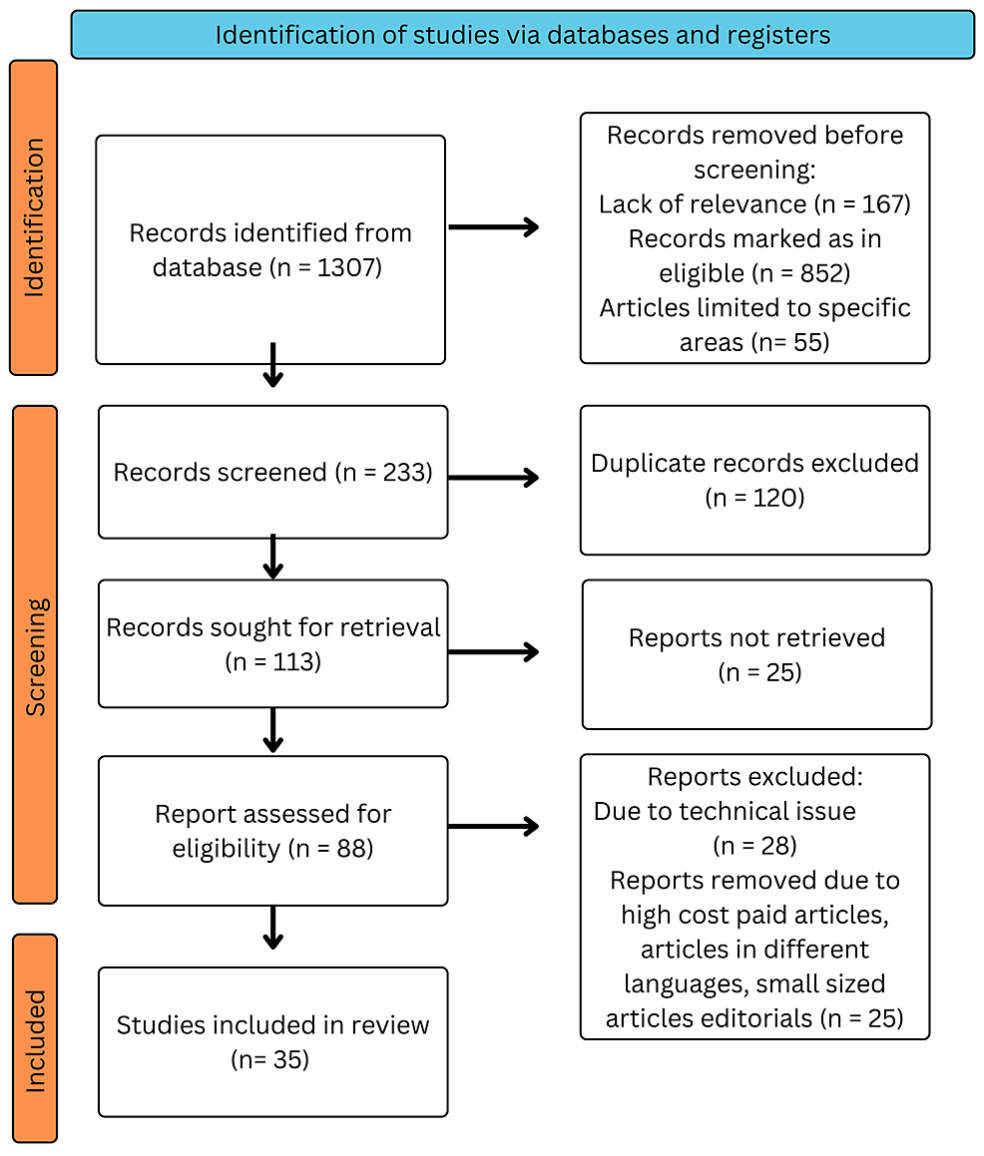
PRISMA methodology for literature search Adapted from the Preferred Reporting Items for Systematic Reviews and Meta-analysis (PRISMA) guidelines. Image credit: Akash Pathak

This comprehensive analysis used a methodical process to compile and examine pertinent research on minimally invasive colorectal surgery techniques. To achieve a thorough grasp of the issue, the study included both qualitative and quantitative research studies and academic articles. To find pertinent materials, a thorough search was carried out across many academic databases, including PubMed, Scopus, and Google Scholar. A database like PubMed was searched using relevant keywords and phrases like "laparoscopy," "robotic," "non-invasive," "colorectal," and "surgery." There were only English-related results shown. The most recent report from a similar study was utilized if there were multiple published reports. We only considered review publications that also included original data. Figure 1 shows the search strategy utilized for the review.

Objective

This research paper aims to conduct a thorough examination of the current status of minimally invasive colorectal surgery, highlighting progress, results, and future outlooks.

Assessing Progress

The main goal of this review is to examine current developments in laparoscopic and robot-assisted minimally invasive colorectal surgical techniques [10]. Our objective is to carefully examine the body of literature to identify the benefits, constraints, and technical issues related to each strategy. We also plan to look at cutting-edge techniques and upcoming technology that might enhance surgical accuracy and patient outcomes.

Evaluating Medical Results

A comprehensive evaluation of the clinical results associated with minimally invasive colorectal surgery is another key goal [11]. We will assess several factors, including postoperative pain, hospital stay length, surgical complications, conversion rates, oncological outcomes, and patient satisfaction, through a thorough study of several papers. We aim to provide a thorough assessment of the advantages and disadvantages of minimally invasive procedures compared to traditional open surgery by employing a methodical approach [12].

Identifying Patient Selection Criteria

Finding the best criteria for selecting individuals for minimally invasive colorectal surgery is the aim of the research we are conducting. We will analyze factors including age, pre-existing health issues, tumor location, and cancer stage to determine which patients would benefit most from less invasive treatments [13]. We will also look into how institutional factors and the surgeon's experience affect patient choice and outcomes.

Exploring Future Perspectives

This review's objectives go beyond just reviewing the state of minimally invasive colorectal surgery today. It also explores potential directions for progress and the prospects for the future [14]. We will research cutting-edge techniques like single-incision laparoscopic surgery and natural orifice transluminal endoscopic surgery (NOTES), coupled with cutting-edge innovations like tele-mentoring and augmented reality [15]. Additionally, emphasis will be placed on ongoing studies and clinical trials meant to improve and expand the use of minimally invasive procedures. This review article's main goal is to give doctors, surgeons, and researchers a comprehensive understanding of the developments, outcomes, and future directions in minimally invasive colorectal surgery [16]. By combining current studies, we hope to advance knowledge, support evidence-based decisions, and promote the use of the best surgical techniques for the well-being of those having colorectal surgery.

Discussion

Robot-Assisted Laparoscopic Surgery for Rectal Cancer: The Ideal Solution?

Robot-assisted laparoscopic prostatectomy is becoming increasingly common. Straight laparoscopic tools have limits in small anatomical areas; however, with EndoWrist instruments and a modern three-dimensional vision system, these restrictions are addressed. Particularly in the extraperitoneal area of the pelvic cavity, surgeons are drawn to the idea of robot-assisted laparoscopic surgery for rectal cancer [17]. However, due to restrictions on unrestricted movement throughout the abdominal quadrants and the higher expense without any discernible advantages over laparoscopic surgery, the use of robot-assisted laparoscopic colectomy is limited in cases of colon cancer. Forty robot-assisted right hemicolectomies were compared to 135 laparoscopic procedures in a retrospective study, and the results showed no appreciable changes in the short-term outcomes of estimated blood loss, conversion rates, complications, or hospital stay [18]. Notably, compared to the laparoscopic group, the robotic group had considerably longer operating durations and higher expenses (p 0.001 vs. p = 0.003, respectively). According to a meta-analysis of seven nonrandomized investigations, there was no discernible increase in the short-term benefits, while the typical operation took 39 minutes longer on average and cost $792 more than traditional laparoscopy. However, it's significant to highlight that robotic surgery, which has a less challenging learning curve, is well-suited for rectal procedures [19]. According to data gathered from 50 robot-assisted laparoscopic rectal surgery cases, the learning curve flattens out after 15 to 25 operations. Unfortunately, expense has prevented this technique from being widely used. The majority of the current research supporting the advantages of robot-assisted laparoscopic rectal cancer surgery comes from case studies. Robotic surgery is both feasible and similar to laparoscopic TME, according to case-matched and nonrandomized studies [20]. To assess if robotic surgery and conventional laparoscopic surgery are equivalent, nevertheless, a sizable prospective, randomized, controlled experiment is required.

Techniques: Total Robotic or Hybrid Approach?

There is continuous discussion over the placement of ports, docking methods, and tactics for controlling splenic flexure during robot-assisted laparoscopic TME. One fully robotic surgery and one hybrid strategy, which differs depending on the tool used for the splenic flexure takedown, are the two main procedures that are primarily employed. Some surgeons use a hybrid technique, depending on a straight laparoscopy for mobilizing the splenic flexure while using a robotic surgical system for vascular control and pelvic dissection [21]. The goal is to shorten the lengthy operation periods caused by several robot dockings. Due to restrictions in port placement and robot positioning, the approach of fully robotic surgery creates difficulties. To finish the task, the robot must frequently dock more than once. A single-stage approach has been looked into by certain centers [22]. However, no published research contrasting alternative docking strategies exists as of yet. The choice of procedure is still greatly influenced by the preferences and experience of the surgeon. In our opinion, there isn't a perfect approach that applies to everyone. It's critical to comprehend the benefits and drawbacks of each strategy, and surgeons must be prepared to customize the surgical approach for each patient [23].

Single-Incision Laparoscopic Surgery

The benefits of conventional multiport laparoscopy (CML) over open surgery are well-known and include less discomfort, shorter hospital stays, fewer wound problems, and a better outward look. By performing the whole process through a single abdominal incision, single port laparoscopic surgery (SILS) can maximize the benefits of CML while minimizing surgical stress. Single port laparoscopic surgery is a development in the direction of scar-free, minimally invasive surgeries. This method deviates from the basic "triangulation" idea of laparoscopy and poses difficulties by containing all laparoscopic ports within a single incision. Compared to CML, SILS requires more advanced laparoscopic skills, is more complicated, has poorer ergonomics and early studies suggest longer operating times than with CML [24]. Beyond CML, SILS has particular technical and ergonomic obstacles, such as the lack of triangulation, conflicts between the camera and instruments, constrained viewing angles, and instrument congestion at the port site. Even for seasoned laparoscopic surgeons, these obstacles provide hurdles. Inadequate dissection and mobilization may result from limited vision and reduced range of motion, increasing the risk of accidental damage. These challenges are magnified in colorectal surgery due to the wider surgical field. The inherent limits of SILS have been addressed in several ways, including the use of curved and articulated instruments, unique SILS ports, and innovative endo-retractors, as well as tailored lengths for the instruments and the camera. Remzi et al. [25] first reported the SILS colectomy in 2008, and following that, colorectal surgeons began to embrace it widely. According to reports, the learning curve for SILS colectomy procedures is between 30 and 60 instances. The length of procedures has decreased as surgeons become more adept at using SILS, which is currently equivalent to that of traditional multiport laparoscopy. Although this can be impacted by reporting bias, considering that SILS is primarily performed by a restricted group of highly trained laparoscopic surgeons, in certain studies, mean operating times for SILS were even reported to be less than those for CML. The SILS strategy has been used to effectively complete the majority of colorectal surgeries, including Hartman's procedure reversal, whole abdominal colectomy, and proctectomy [26]. Numerous randomized clinical studies and meta-analyses contrasting the SILS strategy with CML for colon cancer have been published. The mounting evidence suggests that, in terms of safety and viability, SILS is equivalent to CML. When it comes to things like intraoperative blood loss, bowel function recovery, hospital stay, and incision length, SILS has proven to be superior to CML [27]. Early results indicate that routine laparoscopy and oncological outcomes are also compatible. It's important to remember that the majority of investigations were carried out by skilled and knowledgeable laparoscopic colorectal surgeons. Therefore, it is not necessary to provide a general recommendation for the use of SILS during colorectal surgery. Before SILS for colorectal cancer is widely used, great thought should be given to properly educating mainstream colorectal surgeons in light of the steep learning curve [28].

Learning Curve for Laparoscopic Surgery for Rectal Cancer: Is There a Magic Number?

Rectal resections are more difficult to do than colectomies, as previously indicated, because of the complicated pelvic architecture, the significance of maintaining autonomic nerve function, and the effects of neoadjuvant therapy. Achieving enough exposure is essential in traditional open surgery, especially in male patients with a big mesorectal tumor and a restricted pelvis, where the retractor plays a significant role. For the necessary total mesorectal excision, a precise and crisp dissection is required to produce the best oncologic outcomes. All of these elements work together to make laparoscopic rectal resection more challenging [29]. A Japanese study looked into how proficiency in laparoscopic low anterior excision develops with time. Patients were divided into five groups for the investigation, which encompassed 250 procedures by 21 different surgeons. After the first 50 instances, the surgery time stabilized, according to a review of the learning curve. Notably, after 150 cases (p=0.05), the rate of conversions to standard surgery dramatically decreased, especially for male patients and those with advanced T stages [30]. According to other studies, a sufficient learning curve is often attained in between 20 and 60 instances. In laparoscopic complete mesorectal excision, it's crucial to get the right exposure without using a retractor. As a result, the proficiency of the surgical team as a whole as well as the surgeon becomes essential. The assistant operator may become confused by the camera's reflected image's orientation and the first assistant's tools' alignment in their opposing orientations. One study determined that helping in more than 30 to 40 cases is necessary to become used to the mirror-image motions. The precise number of instances required to achieve technical competency has not been determined by published publications with a strong degree of agreement. A practitioner should successfully conduct at least 20 laparoscopic resections of benign colon lesions before being certified to remove colon cancer, according to data derived from COST research, the American Society of Cataract and Refractive Surgery, and the Society of American Gastrointestinal and Endoscopic Surgeons [31]. The remarkable short-term results of top surgeons in the COREAN experiment, who were in the top third for surgical experience in their individual cancer centers, clearly show that more procedures are linked to better outcomes [32].

This brings up a crucial issue: How can operators get the essential abilities without putting patients in danger? The utility of simulation, animal training, and cadaver courses in appropriately preparing surgeons for resection surgeries on human patients is yet unknown [33]. From our vantage point, a conclusive response to this is still elusive. Any training program, especially one at an advanced level, should place a strong emphasis on selecting wise cases and on intraoperative monitoring by an experienced surgeon. The success of this strategy depends on safe clinical practice. For a comprehensive mesorectal excision using the laparoscopic approach for rectal cancer, specialized and advanced technical expertise is needed. With a very modest conversion rate (11%), the American College of Surgeons Oncology Group (ACOSOG) Z6051 study had one of the highest rates of full or nearly complete total mesorectal excision utilizing the laparoscopic method (92%) [34]. The experiment did not, however, definitively prove that laparoscopy is superior to the open method because of a composite endpoint that lacked statistical validation. However, the authors were able to show that laparoscopy produces better oncological results. The laparoscopic method clearly offers a considerable benefit in the treatment of colorectal cancer, despite the difficulties described. These benefits are extremely important for the healthcare system, especially in light of the rising US national health spending, which has already topped $3 trillion and is anticipated to reach $5 trillion by 2023. Consequently, it is crucial to lower the cost of colorectal surgery by increasing effectiveness, reducing postoperative hospital stays, and lowering post-operative complication rates. These goals are accomplished with a laparoscopic procedure while preserving comparable oncological results [35].

Findings from multiple studies are listed in Table 1.

**Table 1:** Findings from various sources in tabulated format along with the year of publication and country of origin

Authors	Year	Country	Findings
Marusch F et al. [1]	2001	Germany	If we wish to maintain the conversion, morbidity, and mortality rates of laparoscopic colorectal surgeries as low as feasible, we must carefully choose patients based on the surgeon's experience.
Memon S et al. [2]	2012	Australia	Comparing robotic surgery to traditional laparoscopic surgery, the conversion rate was lower.
Weber PA et al. [3]	2002	USA	The feasibility of telerobot-assisted laparoscopic colectomy calls for further study in controlled studies.
Lujan HJ et al. [4]	2018	USA	The tendency for postoperative complications and fatality rates to be lower suggests the safety and effectiveness of the robot-assisted operation.
Park JS et al. [6]	2018	South Korea	Robot-assisted colectomy has similar long-term effects as compared with laparoscopic-assisted colectomy.
Kulaylat AS et al. [7]	2017	USA	A robotic colectomy had decreased conversion rates and a shorter length of stay.
Green BL et al. [9]	2012	UK	There is evidence to support the use of laparoscopically assisted surgery for colonic and rectal cancer, thanks to the extensive period of follow-up reviewed here.
Acuna SA et al. [12]	2019	Canada	Regarding the specific quality of surgical resection outcomes, laparoscopy was comparable to open surgery for the excision of rectal cancer.
Kim HJ et al. [13]	2018	Korea	The robotic approach was associated with reduced disruption of sexual and urogenital functions.
Yamaoka Y et al. [14]	2020	Japan	In lower rectal cancer surgery, robotic assistance may be a helpful strategy to safeguard urine function.
Jayne D et al. [15]	2017	UK	According to research, robot-assisted laparoscopic surgery does not appear to be more effective in removing rectal cancer when carried out by surgeons with different levels of robotic surgery experience.
Yamaguchi T et al. [17]	2018	Japan	Robot-assisted laparoscopic surgery is technically feasible and has good long-term outcomes.
Deijen CL et al. [19]	2015	Netherlands	A robust surgical quality assurance protocol should be applied to maintain the consistency and validity of the clinical trials.
Perivoliotis K et al. [20]	2022	Greece	Similar learning patterns are produced by laparoscopic colorectal procedures in a non-structured training environment compared to the corresponding structured training curves.
Cuschieri A. [22]	2006	Italy	Various error categories exist in surgical settings.
Pitiakoudis M et al. [23]	2011	Greece	An organized training program with a pre-clinical phase emphasizing the acquisition of fundamental skills and a clinical phase emphasizing expert mentorship can reduce the learning curve and enhance clinical results.
Gkionis IG et al. [24]	2020	Greece	After conducting at least 50 different cases using a well-structured and standardized surgical process, a surgeon becomes proficient in laparoscopic colorectal surgery.
Schlachta CM et al. [26]	2001	Canada	Based on a decrease in operating time, intraoperative complications, and conversion rate, this study's learning curve for conducting colorectal resections was about 30 operations.
Choi DH et al. [28]	2008	Korea	The moving average technique may be used to create useful learning curves for laparoscopic sigmoidectomy, which is used to treat sigmoid colon cancer that is curable.
Dinçler S et al. [29]	2003	UK	Measurement of a decrease in operation time should only be one aspect of a learning curve's evaluation; conversion and complexity rates should also be considered.
Bokhari MB et al. [30]	2010	USA	According to the research, a surgeon may become more proficient and contemplate using this procedure on patients who appear to have more complex situations following a learning curve period of 15 to 25 instances.
Manigrasso M et al. [31]	2021	Italy	In order to address several problematic abilities of traditional laparoscopic surgery, robotic surgery has been established.
Nasseri Y et al. [32]	2020	USA	Total operating time and postoperative complication rates were on the decline.
Szymczak P et al. [33]	2021	Poland	After 28 instances, there was stability in the overall operating time for laparoscopic pectopexy based on the Kwiatkowski-Phillips-Schmidt-Shin test.
Lane T. [34]	2018	UK	Newer technological businesses have only recently begun to appear, adding a considerable amount of competition and choice to this quickly developing industry.
Bhandari M. [35]	2020	USA	Despite the enormous rise of autonomous robots, a surgical application for them is still far off.

## Conclusions

The physical restrictions imposed by the bony pelvis and the necessity to preserve autonomic nerves make minimally invasive surgery for rectal cancer challenging. Straight laparoscopic, hand-assisted, and robot-assisted laparoscopic surgery have all been proposed as solutions to these problems. Laparoscopic rectal cancer surgery offers similar short-term benefits to other minimally invasive procedures. According to the available data, survival and recurrence rates for long-term oncological outcomes are comparable. It is anticipated that ongoing prospective, randomized, controlled trials will support the idea that laparoscopic surgery is superior to open surgery. Additionally, it seems that both methods have similar short- and long-term oncological consequences. Robotic surgery seems to promote a faster recovery of sexual and urogenital function. Three less invasive methods of local excision are now available, and they are all as effective as traditional local excision. In conclusion, minimally invasive surgery for rectal cancer has developed into a recognized and well-studied procedure over the past 30 years. However, concerns about the superiority of one modality over another persist, and future technological improvements are anticipated to put the present established platforms under pressure.
